# Parental views on informational counselling provided by audiologists for children with permanent childhood hearing loss

**DOI:** 10.4102/sajcd.v68i1.799

**Published:** 2021-05-25

**Authors:** Zandile M. Shezi, Lavanithum N. Joseph

**Affiliations:** 1 Department of Audiology, Faculty of Health Sciences, University of KwaZulu-Natal, Durban, South Africa

**Keywords:** informational counselling, communication options, aural rehabilitation, family-centred intervention, paediatric hearing loss

## Abstract

**Background:**

The absence of best practice guidelines on informational counselling, has caused lack of clarity regarding the information audiologists should provide to parents and caregivers following the diagnosis of a hearing loss. Research shows that informational counselling provided by audiologists is limited and often biased, with little evidence of how parents experience this service.

**Objectives:**

To explore the nature and practice of informational counselling by audiologists.

**Method:**

This study was descriptive in nature and adopted a survey design to obtain information on the current practices of informational counselling from the perspective of parents and primary caregivers. Ninety-seven face-to-face semi-structured interviews were conducted across KwaZulu-Natal province of South Africa. Descriptive statistics and thematic analysis using Nvivo software were conducted.

**Results:**

The majority of the parents reported receiving some form of informational counselling. However, the information provided by audiologists was considered to be biased as it included a favoured communication option, school and rehabilitative technology. There was a lack of information related to aural rehabilitation and family-centred intervention. The provision of all communication options, school options and rehabilitative technology were identified as gaps that contribute to an unfavourable decision-making process.

**Conclusion:**

There are inefficiencies experienced by families of deaf and hard of hearing children during informational counselling. However, this understanding, together with the identified gaps by parents, can help address the professional response to caring for families with deaf and hard of hearing children.

## Introduction

It is reported that 90% – 92% of children who are deaf are born to hearing parents who have little or no experience of deafness (Mitchell & Karchmer, 2004; National Deaf Children’s Society, 2015). Experiencing deafness for the first time, these parents have no knowledge about how to manage the intricacies presented by the hearing loss. When deafness is confirmed, parents rely on professionals to provide them with information about the intervention options that are available for their child (American Speech and Hearing Association [ASHA], 2008; Gilliver, Ching, & Sjahalam-King, 2013; Tye-Murray, 2019) and this process is conducted through informational counselling. Informational counselling can be defined as the dissemination or impartation of information that relates to but is not limited to the interpretation of the audiogram, and deciding on technology, educational and communication options (ASHA, 2008). Currently, there are no evidence-based studies that provide information that parents require from audiologists to make informed decisions about intervention.

Audiologists need to provide families with information that will assist them in the decision-making process; however, this is often a challenge. It has become common practice for audiologists to filter a substantial amount of information, which they should present to parents during informational counselling (ASHA, 2008). Extensive research conducted in the United Kingdom has shown that only after engaging with certain intervention services did parents realise that the information provided by professionals regarding available options to support linguistic and social development for their children who are deaf or hard of hearing was limited (Beazley & Moore, 1995; Eleweke & Rodda, 2000; Young, 2003; Young, Jones, Starmer, & Sutherland, 2005), indicating that informational counselling provided by audiologists is limited and biased. However, there is a lack of evidence in the literature about the actual experiences of parents regarding informational counselling provided by audiologists. Current literature does not address the gaps that parents have identified in informational counselling provided by audiologists following diagnosis of the hearing loss. In addition, there is scarce literature regarding the factors that audiologists need to consider when providing informational counselling, that is, factors that are relevant and unique to these families, which will influence how informed decision-making occurs related to intervention options and how the informational counselling will be perceived. This situation, together with the unique challenges experienced by parents in countries with huge linguistic, cultural and socio-economic diversity such as South Africa, led to the interest in this study.

### Literature review

According to the literature, a parent’s primary concern relates to communication choices (Young, Carr, Hunt, Skipp, & Tattersall, 2006). The issue was not so much that a particular communication option was right or wrong, but the issues stemming from three positions. Firstly, if parents do not have access to all relevant information required to make informed decisions, the appropriateness of the basis on which such decisions are made is questionable. Secondly, the information provided to parents did not provide all available options because it was either denied, not acknowledged or resourced. Lastly, if the professional’s attitude and bias dominates, this influences the potential of the parent–professional relationship to be one of empowerment (Young et al., 2006). According to Young (2002), parents reported that they were predominantly presented with the medical model of deafness and in hindsight were exposed to the socio-cultural model and other approaches. Another domain, although not well researched, was the aspect of deaf parents who were not provided with all options. This was attributed to either the assumption that they would not require certain information because they were deaf or the information could not be linguistically communicated to them (Young et al., 2005). It must be pointed out however that the audiologist is responsible for the facilitation of informed decision-making across the clinical care process and journey (Oestreich, 2019).

According to English (2008), there still remains a need for effective informational counselling to be considered in the body of audiological literature. According to Watermeyer, Kanji and Cohen (2012), informational counselling guidelines are non-existent for the audiology population. The following suggestions have been made on how informational counselling should be carried out as a service to patients (Margolis, 2004; Musiek, Weihang, & Oxholm, 2007): for the benefit of decision-making, the information needs to be adequate, clear and concise; concrete instructions should be explicitly provided; important information should be provided first; information needs to be repeated; the terminology used should not mislead and confuse the patient with verbal descriptions supplemented by an anatomical model or diagram depicting the ear. During informational counselling, cultural diversity, linguistic barriers and inclusion of a third party should be considered (Watermeyer et al., 2012). Language barriers can contribute to diminished access to information related to primary and preventative care. This barrier can contribute to the patients’ weakened comprehension ability, minimise adherence and cause reduced patient satisfaction (Wilson, Chen, Grumbach, Wangs, & Fernandez, 2005). The reality in South Africa is that there are 12 official languages and diverse cultural beliefs and healthcare interventions are centred within these unique linguistic and cultural barriers (Penn, 2007). Also, a typical South African patient accesses traditional healers parallel to biomedical options (De Andrade & Ross, 2005). These factors have implications for diagnosis and how intervention is carried out regardless of the informational counselling received. In South Africa, the average age of diagnosis is 2 and a half years and this is considered to be late (Van der Spuy & Pottas, 2008). Late diagnosis affects the cognitive, communication, social and educational development of this child (Yoshinaga-Itano, Sedey, Coulter, & Dehl, 1998), which makes the process of decision-making for the parents even more complicated and urgent. With so many children diagnosed late with a permanent childhood hearing loss in South Africa, immediate and urgent intervention is required to combat the developmental gap created by this delay. This then requires parents to make important intervention decisions and this process is dependent on the information or intervention options that parents receive. The addition of limited and biased informational counselling exacerbates this challenge of hearing loss particularly in the South African context and contributes to a further delay, thus prohibiting any opportunity of minimising or closing the developmental gap. Although extensive research has been conducted about informational counselling being viewed as biased and limited, not much is known about the actual current practices of informational counselling provided by audiologists.

The purpose of this study was to explore the practices of informational counselling provided by audiologists when children are diagnosed with a permanent hearing loss, with a view to identify information needed by parents or primary caregivers to make informed decisions regarding intervention and factors that need to be considered when providing informational counselling.

The research question posed was the following: ‘what is the nature of the informational counselling provided following the diagnosis of a permanent childhood hearing loss?’.

Thus, the study aimed to determine the practices of informational counselling provided by audiologists in KwaZulu-Natal (KZN) province in South Africa in the past 5 years, identifying gaps in informational counselling and exploring contextually relevant factors that audiologists need to consider during informational counselling with parents and primary caregivers of children who are deaf or hard of hearing.

## Research methods and design

The study was descriptive in nature with both qualitative and quantitative components. A descriptive survey was conducted using face-to-face interviews. An interview schedule with open-ended questions was used.

### Study population

The participants were hearing parents and primary caregivers of children who are deaf or hard of hearing residing in KZN province. This was done to facilitate homogeneity of the participants looking at the region. The parents and primary caregivers were accessed from 31 hospitals within all 10 districts representative of KZN providing paediatric audiology services.

### Sampling method

To recruit the participants, random sampling was employed. Patient files were accessed from the public hospitals and contact numbers were retrieved. Participants who met the selection criteria were contacted telephonically and were addressed in English or isiZulu by the researcher who is a first language isiZulu speaker. For participants who were non-literate, the study was verbally explained to them. The participants were contacted to provide details regarding the study, and to schedule a convenient date, time and place to conduct interviews. Private audiologists made contact with potential participants regarding the study. Interested participants contacted the researcher. The researcher randomly selected parents and primary caregivers from all public hospitals (80) and private clinics or hospitals (17) using the KwaZulu-Natal Department of Health database. Limited participation from some private facilities was attributed to paediatric audiology services not being offered and some audiologists withholding access to potential participants on their caseload.

### Sample size

A statistician was hired to ascertain the sample size. A sample size of 97 randomly selected parents produced a two-sided 95% confidence interval with a width equal to 0.20 or 20% (i.e. ± 10%) when the sample proportion is 0.50 or 50% (non-informative assumption, assumes maximum variability) (Fleiss, Levin, & Paik, 2003; Hintze, 2013; Newcombe, 1998). A total number of 97 parents and primary caregivers were interviewed.

### Description of the sample

The sample included 1 (1%) father, 40 (41%) mothers and 56 (58%) primary caregivers (grandparents, siblings or aunts). A total of seven (14%) parents were first language English speakers, with the remainder being first language isiZulu speakers. The age range of participants was between 18 and 62 years. The level of education of the participants ranged between grade 2 and an honours degree. All participants had children who were deaf or hard of hearing diagnosed within the past 5 years in 31 public hospitals and three private clinics within KZN and had an age range between 8 months and 9 years.

### Data collection instrument

The data collection instrument, that is, interview schedule, was derived from the literature taking into consideration the research question. The interview schedule comprised a demographic section and open-ended questions related to the age of diagnosis, type of counselling received, the content areas covered during informational counselling, grieving process, identified gaps in informational counselling, factors audiologists need to consider when providing informational counselling, family-centred intervention, the decision-making process and views on the informational counselling received. Face-to-face semi-structured interviews were conducted by the researcher in either English or isiZulu. The duration of the interviews ranged from 10 min to 37 min and the interviews were audio recorded.

### Procedure

A pilot study was conducted on 12 participants and data obtained during this process were not used in the main study. Changes were made to the revised interview schedule. The interviews were audio recorded using the Olympus digital voice recorder DM-650, Olympus Audio SA and transcribed by a professional transcribing company. Thematic analysis using Nvivo 12 was conducted to process and analyse the data. All interviews in isiZulu were transcribed into English and then backtranslated by a professional company. These were then verified by the researcher before being uploaded into Nvivo for analysis.

### Ethical considerations

All ethical considerations were adhered to in this study. Issues related to informed consent, voluntary participation, withdrawal and anonymity were addressed. Ethical clearance was obtained from Biomedical Research Ethics Committee of the University of KwaZulu-Natal (Ethical clearance number: BE267/16).

## Results

### Experience of informational counselling

Of the 97 participants interviewed, six (6%) participants reported that they did not receive any informational counselling from the audiologist or any other health professional, whilst two (2%) could not recall if they had received any informational counselling. The remaining 89 (92%) participants reported to have received some form of informational counselling; however, the content areas addressed and the manner in which the information was provided during these sessions varied for all participants.^1^

One participant stated:

‘No, we were never counselled, she just got tested and they diagnosed her with hearing loss and they said she will get a hearing aid and they made an appointment for us to come and get the hearing aid.’ (P1, granny, public sector)

**FIGURE 1:**
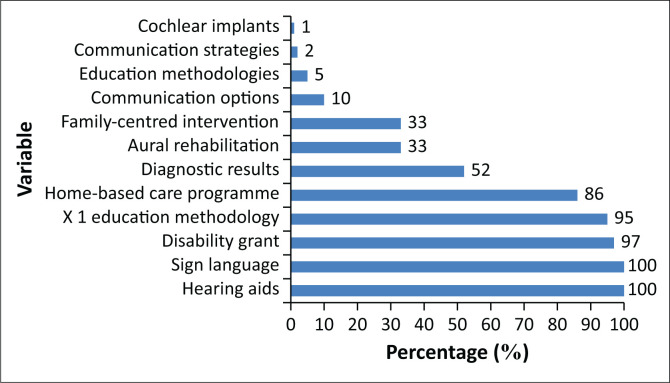
Content areas discussed during informational counselling (*n* = 89).

Figure 1 presents the content areas that were covered during informational counselling (*n* = 89).

All 89 (100%) participants who were counselled reported to have received information about hearing aids and South African Sign Language as the only communication option mentioned. Only nine parents of the 89 who received counselling (10%) reported that the audiologist counselled them about all the available communication options, such as total communication, oralism and bilingual–bicultural, and only four (4%) reported to be informed about more than one education methodology. Two parents (2%) reported receiving information about communication strategies and only one (1%) parent was reported to be informed about cochlear implants as an available rehabilitative technology. A total of 76 (85%) of the 89 parents or primary caregivers reported that the parent advisors from a national non-profit organisation offering a home-based care programme provided detailed information; however, this information was only on Sign Language and the bilingual bicultural education methodology. These parents reported that although the information was extensive, they would have preferred extensive information on other communication options as well. These parents also reported to have benefitted from the basic Sign Language lessons provided by the representative from the home-based care programme, as evident in the following quotes:

‘They gave me the information based on their views.’ (P2, mother, private sector)

‘There is nothing else they explained to us, they just explained about the sign language.’ (P3, mother, public sector)

The majority, (85, 88%) of these parents or primary caregivers reported to be informed about the availability of a disability grant, whilst almost the same number (84, 87%) reported to have been informed about one education methodology, that is, bilingual–bicultural school for the deaf. Whilst 46 (47%) parents or primary caregivers reported information about the diagnostic results, only 29 (30%) reported counselling about aural rehabilitation and the significance of family-centred intervention and the implications of the hearing loss in terms of communication.

### Perception of gaps in informational counselling

**FIGURE 2:**
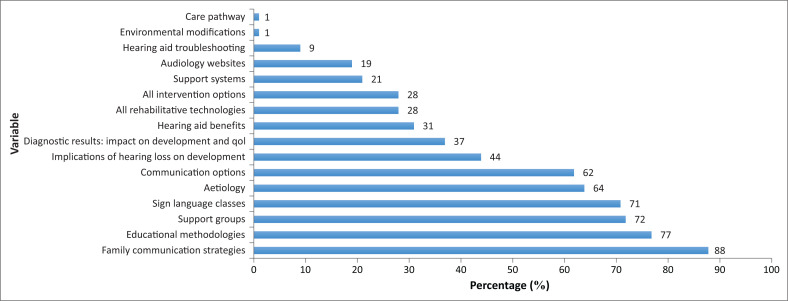
Information that parents deem critical for intervention decision-making (*N* = 97).

The identified gaps represented information that parents deemed critical for intervention decision-making, which was either absent, limited and/or biased during the informational counselling they received. These content areas are presented in Figure 2.

More than 50% of the participants reported that informational counselling needs to include aetiology of the hearing loss, all education methodologies, parent-to-parent support groups, all communication options, family communication strategies and Sign Language classes. About 30% – 45% of the participants in the study reported that there are gaps in information related to implications of the hearing loss, diagnostic results and hearing aid benefits. The remaining content areas identified as gaps were considered by less than 30% of the participants.

The number of participants in relation to the content areas identified as gaps was based on the information that each participant deemed critical based on whether they did or did not receive it. A total of 64% of participants reported that audiologists need to provide information on the cause of the hearing loss, as shown in the following quotes:

‘I can say at the hospital, we got information, but something we never got was what the cause was for her hearing loss. That it was natural or what.’ (P4, father, public sector)

‘The only suggestion I have is that as a mother of the child, I was all alone and the father left me after finding out that the child has a hearing loss. They were supposed to tell me what the reason is, what the reason for my child losing her hearing was this and that.’ (P5, mother, public sector)

A total of 77% of participants reported that not all education methodologies were provided. The majority of these participants were reported to be informed of one education methodology and only later found out about other education methodologies, as clear from the following quote:

‘They advised me about special schools only, they never spoke, explained the results. No they didn’t explain in detail and the cause you see?’ (P6, mother, public sector)

Parent-to-parent support groups were reported as a gap during informational counselling by 72% of the parents and primary caregivers:

‘I wanted to know from other people that how did they react to it when it happened to their kids.’ (P7, mother, private sector)

A total of 62% of parents or primary caregivers reported that the provision of all communication options was a gap. A total of 88% of parents or primary caregivers reported gaps in the provision of information about communication intervention strategies, whilst 71% of these parents or primary caregivers reported Sign Language classes as a gap during informational counselling. The impact of the hearing loss on speech and language development was reported as a gap by 44% of the parents or primary caregivers. Amongst other reported gaps by participants were care pathways (1%), hearing aid benefits (31%) and troubleshooting (9%), websites (19%), support systems (21%), environmental modifications (1%) and all available rehabilitative technologies (28%).

### Aspects to consider during informational counselling

**FIGURE 3:**
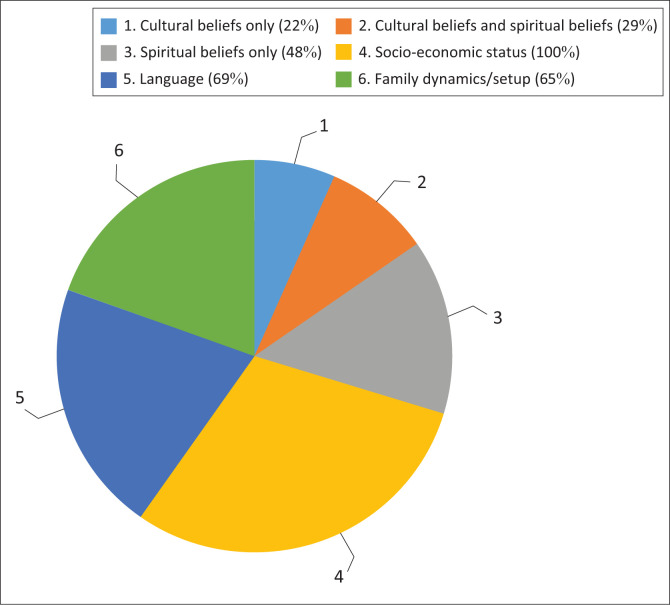
Aspects to consider during informational counselling.

Some parents and caregivers reported that for informational counselling to be relevant and influence compliance for follow-up sessions, various aspects need to be considered and evident during informational counselling. Figure 3 depicts the aspects that need to be considered during informational counselling.

Twenty-one (22%) parents and primary caregivers reported that when providing informational counselling, audiologists need to factor in their cultural beliefs only, whilst 28 (29%) parents and caregivers stated that a combination of spiritual beliefs and cultural beliefs needed to be considered during informational counselling. Interestingly, 48 (49%) parents and caregivers reported that audiologists had to factor in their spiritual beliefs as opposed to culture when providing informational counselling. All parents (100%) in the study reported the significance of considering the socio-economic status during informational counselling. As the majority of the parents were first language isiZulu speakers, 67 (69%) proposed that audiologists consider the language used during the provision of informational counselling. Furthermore, 63 (65%) parents reported the significance of considering family dynamics in the provision of informational counselling.

## Discussion

This study revealed that the majority of parents were only informed about hearing aids and the bilingual–bicultural communication option during informational counselling. These findings concur with the study by Duldulao and Ramsey (2013), who reported that it is common practice for audiologists to place emphasis on providing hearing aids as opposed to communication intervention despite aural rehabilitation being considered a significant component in the management of hearing loss. According to these authors, with rapid advances in hearing technology, technology alone is not adequate to address the hearing needs of individuals with hearing loss. In addition, Prendergast, Lartz and Fiedler (2002) discovered that more than half of the parents in the study were not told about more than one communication option. Similar to the findings of this study, the professionals included in the study by Young (2002) overtly sided with a particular communication method and provided detailed information on their preferred communication method, hence neglecting other communication options. The ease of access and availability of a communication method could be attributed to such practices, although it is not justified. Provision of one communication option could lead to one education methodology as there is a link between communication options and education methodologies. For this study, the informational counselling on one communication option could be attributed to the ease of referring these families to schools that adopt the bilingual–bicultural approach for the child to acquire South African Sing Language (SASL) as opposed to providing auditory verbal therapy or total communication services. The SASL is defined as a language used by many individuals in the Deaf community. It is the primary language of Deaf South Africans with its own grammar, structure and syntax which differs from the spoken language (DeafSA, 2006). These findings may also speak about the availability and accessibility of certain schools or aural rehabilitation services within the contexts of the families. Additional to these issues could be issues related to the age of diagnosis and its influence on the developmental gap in speech, language and communication. Literature reveals that when parents select a school, they consider cost, geographic location and benefits of enrolling in that school (Calderon, 2000). When only one education methodology is presented to families, this minimises the choice and infringes on informed decision-making.

In this study, a great number of parents and caregivers were exposed to information regarding the disability grant. Whilst it is commendable that audiologists are providing these families with the opportunity to access these funds for the benefit of the child with the disability, audiologists should not lose sight of the ethical benefits of this grant and their role in the rehabilitation process to ensure independent individuals in the future. This grant should not be used as a substitute for rehabilitation; however, it should be used in conjunction with rehabilitation to assist families and children who are deaf or hard of hearing to be independent, employable South African citizens. This notion does not disregard the socio-economic status of the majority of these families and the role of the disability grant; however, it supports focus on these children being rehabilitated. Interestingly, the majority of parents were not informed about aural rehabilitation. This questions our stance as rehabilitation specialists, the availability and accessibility of these services and our perceptions of our role as audiologists. These results question the difference between our scope of practice and that of other hearing service providers such as acousticians. The audiologist’s role goes beyond that of diagnosis and extends to that of rehabilitation; however, the results of this study contradict this role in some contexts within KZN province. According to the HPCSA (2012), audiologists are expected to provide aural rehabilitation. Duldulao and Ramsey (2013) reported that whilst some patients may not be inclined to being participants in aural rehabilitation sessions, the informational counselling around aural rehabilitation as a service should be provided, as well as the benefits of aural rehabilitation.

In this study, the participants reported that there was a lack of information regarding the cause of the hearing loss. The lack of providing the cause of the hearing loss by audiologists could be attributed to uncertainty and limitations in the scope of practice as ear, nose and throat (ENT) specialists are expected to provide this information. The fact that some parents blame themselves or are blamed by their spouses when the child is diagnosed with a hearing loss makes the aetiology pertinent for these caregivers. It is when the cause of the hearing loss is known that some parents can deal with the emotions that are aroused by the confirmation of the hearing loss. This also contributes to how parents address and manage the implications of the hearing loss thereafter in relation to traditional beliefs.

The participants in this study stated the need for parent support groups. This was also reported by the participants in the study by Jackson, Wegner and Turnball (2010) as one-third of the parents (*N* = 73) reported to have participated in some kind of parent support group. The common theme identified in this study was the desire for the parents to have access to a parent-to-parent support network. This was a similar finding in this study. Engaging with ‘similar others’ created a sense of belonging for new parents of children who were deaf or hard of hearing and also reduced anxiety that comes as a result of the diagnosis of hearing impairment (Zaidman-Zait, 2007). The parent-to-parent support allows parents to identify the reality of what it is like to have a child who is deaf and hard of hearing and address how parents can make decisions regarding intervention. This may reduce delays in that parents may not try out all available options before making an informed decision, for example, regarding communication options, schooling and rehabilitative methodologies (Narr & Kemmery, 2015). According to Lederberg and Golbach (2002), you need to be a parent of a deaf or hard of hearing child to comprehend the depth of parenting a deaf or hard of hearing child.

For the participants in this study, as ancestral beliefs and religious beliefs are part of who they are, some parents felt that approaching the hearing loss from a medical and cultural model (inclusive of cultural and spiritual beliefs) (De Andrade, 2015) would put them at ease of knowing that they had explored all available avenues to try and support their child who is deaf or hard of hearing. In addition to the combination of the medical and cultural model, the parents and primary caregivers in this study felt that the inclusion of financial implications during informational counselling for parents to be aware of and be prepared for the upcoming expectations on their part was critical. This would facilitate improved adherence and compliance rates to follow-up sessions and intervention options. The socio-economic status is able to influence how the speech and language may develop (Fernald, Marchman, & Weisleder, 2014) and accessibility to hearing rehabilitation (Mehra, Eavey, & Keamy, 2009). Chang, Ko, Murray, Arnold and Megerian (2010) reported that because of low socio-economic backgrounds of some families in Ohio, the United States of America, there was an increased non-compliance rate to follow-up sessions.

Another aspect was the parents of children who are deaf or hard of hearing being counselled by audiologists who spoke a language different from theirs. The profession of Audiology and Speech Language Therapy is dominated by white therapists who speak English and Afrikaans, whilst the client base is dominated by black individuals who adopt African languages (eds. Moonsamy & Kathard, 2015). This mismatch has been attributed to the history of colonialism and apartheid; however, 26 years into democracy, little change has been observed regarding the demographics of these professions. Additional to this aforementioned issue is the recent increase in migration of individuals from other African countries into South Africa, which increases the diversity even more of the potential client base. The majority of clinicians were reported to be providing services to clients who do not use English as a home language (eds. Moonsamy & Kathard, 2015).

### Strengths and limitations of the study

The sample size was a strength of this study as it allowed the researcher to obtain in-depth information that allows for generalisability within the region. The disproportion in the number of participants accessed from the public and private sectors was a limitation of the study.

### Implications and recommendations for future research

This study highlighted contextually relevant factors that need to be considered during the facilitation of informational counselling that contributes to the relevance and decision-making process. Future research may explore the outcome of including contextually relevant factors and how this facilitation may contribute to the decision-making process of parents and caregivers.

## Conclusion

As a result of the controversies and conflicting views on education methodologies, communication options, medical treatment and sensory devices, parents need to be appropriately counselled so that they can be equipped to make informed decisions about the controversial issues that surround intervention for a deaf or hard of hearing child. The information provided for these families through counselling needs to be balanced and at the proficiency level and rate at which family members can absorb the information whilst coping with their emotional reactions. This study highlighted the inefficiencies and lack of service experienced by families of deaf and hard of hearing children. The participants identified informational counselling needs to include the cause of the hearing loss, all available communication options, educational methodologies, rehabilitative technologies and available parent support groups. Audiologists also needed to consider the socio-economic status of the family, cultural and spiritual beliefs and language during the facilitation of informational counselling. This will influence the relevance of informational counselling offered. An understanding of the scope of counselling by audiologists, together with the identified gaps by parents themselves, can help to address the professional response to caring for families with deaf and hard of hearing children, as well as promote parents as advocates for their children.

## Footnotes

1 For the purpose of this article, participants have been given an identifier code P1–P7, as different participants were selected and reported only once.
